# Factors influencing intentions to transition to plant‐based protein diets: Canadian perspective

**DOI:** 10.1002/fsn3.4436

**Published:** 2024-09-12

**Authors:** Gumataw Kifle Abebe, Mariam R. Ismail, Kathleen Kevany, Hiwot Abebe Haileslassie, Liam Young, Treasa Pauley

**Affiliations:** ^1^ Department of Business and Social Sciences, Faculty of Agriculture Dalhousie University Truro Nova Scotia Canada; ^2^ Department of Agricultural, Food and Nutritional Science, Division of Human Nutrition University of Alberta Edmonton Alberta Canada; ^3^ Eastern Canada Oilseeds Development Alliance (ECODA) Charlottetown Prince Edward Island Canada

**Keywords:** Canada, consumer behavior, plant‐based protein foods, sustainable diet, TPB

## Abstract

There is a pressing need for healthy diets guided by environmental and nutritional targets. Plant‐based proteins have emerged as a recent and rapidly growing trend in response to the challenge of sustainable and healthy food systems. While plant‐based protein foods are widely promoted as sustainable alternatives, shifting beliefs and attitudes about conventional protein sources present an ongoing challenge. The study examined Canadians' intentions to transition to plant‐based protein diets, partially or entirely. A nationally representative survey was conducted among Canadian consumers to achieve our research objective. The survey was administered online using the Qualtrics platform by a market research firm and yielded valid responses from over 1800 participants. The Theory of Planned Behavior (TPB) constructs—attitudes, self‐efficacy, and perceived availability—explained only 12% of the variation in intentions toward plant‐based protein foods, while sustainability and ethical concerns accounted for 10% of the variation in dietary patterns. Meat attachment negatively impacted changes in dietary patterns, explaining 11% of the intention variation. Additionally, individual past behavior accounted for 7% of intentions toward plant‐based proteins. Demographic factors, such as gender and education, strongly and positively predicted purchase intentions, while contextual factors, such as residing in rural neighborhoods and being from Atlantic Canada, showed a strong negative association with intentions toward plant‐based protein diets. The findings underscore the multifaceted nature of individuals' intentions toward plant‐based protein diets and emphasize the significance of considering cognitive, social, emotional, and past behavioral factors, alongside sustainability values and messaging, to transition to a more plant‐based protein diet. This approach should carefully balance individuals' emotional connection and the perception of meat as essential to their meals. Also, targeting interventions based on demographic characteristics, specifically gender, education, and residential neighborhood, can enhance changes in dietary protein sources. The findings contribute to the existing body of knowledge on consumer behavior and sustainable diets, guiding future research and policies informing the design of effective interventions to promote plant‐based protein consumption and dietary changes.

## Graphical abstract

The study examined Canadians' intentions to transition to plant‐based protein diets, drawing from primary data from a nationally representative sample of 1800 participants. The findings highlight that achieving the desired transition to sustainable protein diets requires a multifaceted approach that bolsters positive attitudes, self‐efficacy, and availability while emphasizing sustainability and ethical considerations. The insights contribute to understanding consumer behavior and sustainable diets, informing future research and policy interventions to promote plant‐based protein consumption and dietary shifts.

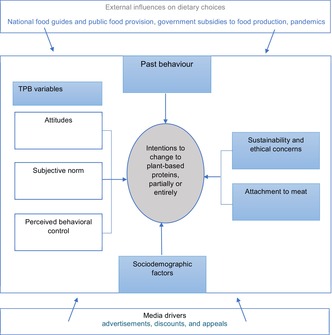

## 1 INTRODUCTION

The global food system is a major contributor of greenhouse gas (GHG) emissions (Crippa et al., 2021). To address the challenges of human and planetary health, it is crucial to foster more resilient food systems that ensure food availability, accessibility, and nutritional adequacy. There is a pressing need for healthy diets guided by scientifically defined environmental and nutritional targets and a diverse range of sustainable protein foods (Kraak & Stanley, 2023; Reisch et al., 2013; Willett et al., 2019). Plant‐based proteins^1^ have emerged as a rapidly growing trend (Aschemann‐Witzel et al., 2021; Langyan et al., 2022; van Vliet et al., 2020). This trend is expected to continue as the food industry continues to innovate and expand its offerings, mimicking the taste, texture, and nutritional profile of traditional protein sources (Boukid, 2021). While plant‐based protein foods are widely promoted as sustainable alternatives to conventional protein sources, shifting beliefs and attitudes about conventional protein sources present an ongoing challenge and are highly context‐specific. Many individuals hold deep‐rooted beliefs and preferences for traditional animal‐based proteins and have misconceptions surrounding the nutritional adequacy and taste of plant‐based proteins (Clark et al., 2022; Day et al., 2022; Santo et al., 2020). As the need to change dietary patterns, wholly or partially, becomes more urgent, it is critical to have a comprehensive understanding of the factors shaping consumers' attitudes and behaviors in embracing plant‐based protein foods as a regular diet.

Against this backdrop, this paper aims to analyze the dynamics in food consumption behaviors, particularly concerning recent changes in intentions toward plant‐based protein foods and the factors influencing their dietary behaviors from a Canadian perspective. Specifically, this study examines Canadians' intentions to increase the consumption of plant‐based protein foods to replace traditional protein sources, partially or entirely. Expanding our understanding of consumer consumption trends in different periods and geographical locations can inform policymakers and the plant‐based protein industry to appeal to the broader segments of society toward sustainable consumption behaviors (Aschemann‐Witzel et al., 2021; Kamenidou et al., 2019; Kemper, 2020; Marcus et al., 2022). Also, understanding the dynamics of consumer behavior and underlying factors that influence their choices is crucial for the broader adoption of sustainable food options. Our study is particularly significant, given the limited national research conducted in the Canadian context (Clark & Bogdan, 2019). The study applies the extended Theory of Planned Behavior (TPB) (Ajzen, 1985). While previous studies have extensively explored the application of TPB in understanding purchase intentions, there is a notable gap in addressing the intersection of TPB and plant‐based protein sources. Only a handful of studies have applied the TPB framework to understand intentions toward plant‐based proteins (de Gavelle et al., 2019; Drolet‐Labelle et al., 2023; Jang & Cho, 2022; Marcus et al., 2022; Seffen & Dohle, 2023). This study broadens the applicability of the TPB model within the specific domain of plant‐based protein foods by incorporating various variables, such as sustainability and ethical concerns, meat attachment, and contextual factors, to predict intentions beyond the traditional TPB constructs (Ajzen, 1991). Our study deliberately focused on intentions to transition to plant‐based protein diets in general rather than focusing on any specific plant‐based protein source. This is because the absence of a preference for a particular plant‐based protein food may not necessarily imply an individual's overall intention to avoid consuming other plant‐based protein foods. This way, our study captures the broader spectrum of purchase intentions toward plant‐based proteins, recognizing that preferences within specific categories may not fully reflect one's intentions to embrace a wider variety of plant‐based protein sources.

## 2 CONCEPTUAL FRAMEWORK

The Theory of Planned Behavior (TPB) (Ajzen, 1991) is a popular framework for predicting human behavior and providing insights into the cognitive processes influencing individual intentions and purchase decisions. Extensive evidence supports TPB's applicability in food consumption (Pandey et al., 2021; Rana & Paul, 2017; Wang & Scrimgeour, 2021). TPB helps explain why individuals make certain food choices and how their intentions align with their behaviors based on three primary factors: attitudes, subjective norms, and perceived behavioral control. The TPB framework offers valuable insights into the underlying mechanisms that shape sustainable food consumption behaviors, facilitating the development of effective interventions and policies to promote sustainable dietary practices. TPB recognizes the importance of behavioral intentions in predicting behavior (Ajzen, 1991), including food consumption behaviors (Vermeir et al., 2020).

In the study context, attitudes toward a plant‐based protein food refer to any positive or negative evaluation of consuming plant‐based protein foods, including beliefs about the health benefits and environmental impact (Bryant & Sanctorum, 2021). Individuals with more positive attitudes toward plant‐based protein food are more likely to have the intention to embrace it as a regular diet (Graça et al., 2015). Subjective norms refer to perceptions of social pressure to engage in a behavior, including the influence of family, friends, and cultural or societal norms. If individuals perceive that adopting a plant‐based protein food is socially desirable within their environment, they may be more likely to have the intention to adopt it (Menozzi et al., 2017). Also, perceived behavioral control may refer to individuals' beliefs in their ability to perform the behavior. This can include practical considerations, such as access to plant‐based foods, cooking skills, and difficulty transitioning to a plant‐based protein food. If consumers perceive that they have control over their ability to adopt a plant‐based protein food, they may be more likely to have the intention to increase the consumption of plant‐based protein foods to replace traditional protein sources, partially or entirely (Drolet‐Labelle et al., 2023).

**FIGURE 1:**
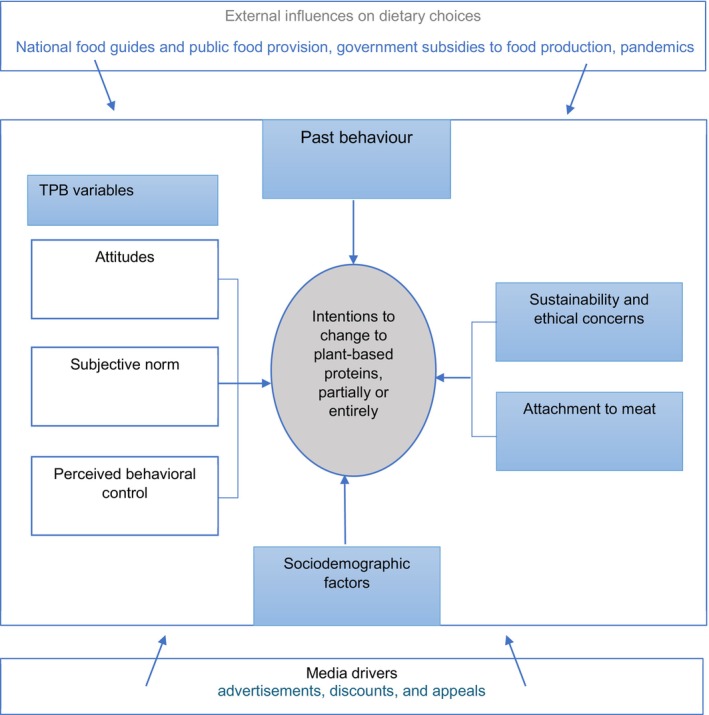
Conceptual framework: Factors predicting intentions to change toward plant‐based protein foods.

While TPB provides a valuable framework and has been successful in understanding and predicting certain human behaviors, it is essential to recognize that it may not encompass all the predictors of more complex consumer behaviors (Chen, 2017; Choi & Johnson, 2019; Contini et al., 2020; Dorce et al., 2021). Food choices can be influenced by a wide range of factors beyond the cognitive components emphasized by the TPB (Carfora et al., 2019; Chen, 2017; Contini et al., 2020; Dorce et al., 2021; Menozzi et al., 2015). The present study expands on the TPB framework by incorporating additional variables predicting individuals' intentions to change their dietary patterns toward plant‐based protein foods. The extended TPB framework recognizes that intentions to change dietary patterns are shaped by a diverse range of variables, including the core components of the TPB. In addition to these fundamental factors, this study acknowledges the influence of other key determinants, such as sustainability and ethical concerns, attachment to animal protein foods, past behavior, and sociodemographic characteristics, including contextual factors, such as geographic location and residential neighborhoods (Figure 1).^2^

## 3 METHODS

### 3.1 Study design and sample

This study is part of a larger project to explore the value of plant‐based proteins in Atlantic Canada. A market research firm, Angus Reid, carried out a quantitative survey among its panel of active members. A stratified random sampling method was used to have proportional sample representations from all regions and demographic groups across Canada. The respondents were recruited randomly from the panel and were balanced for age, gender, and geography. Selection criteria included participants living in Canada and a minimum age of 18 years. An additional effort was made to include more participants from the Atlantic Provinces, as response rates from this region have traditionally been low.

The survey was pilot‐tested with a representative of the sociodemographic population group to assist with construct and content validity and the general understanding of questions. A statement regarding the confidentiality and anonymity of the data was provided before the beginning of the survey. Participants were informed about the study's objectives, the intended use of their data, and their right to withdraw from the questionnaire before submitting their responses. The survey was online (Qualtrics) and carried out in August 2022. It took, on average, 20 to 25 min to complete. The questionnaire was available both in English and French, and translations were provided by Angus Reid.^3^ This study was granted clearance from the University's Research Ethics Board.

We obtained over 1800 valid responses. However, the number of valid responses considered varied in each analysis, specifically in the regression analysis, as some respondents skipped certain questions while completing the survey. The regression analyses only considered respondents with complete responses (*n* = 1557). We acknowledge that this may introduce a potential bias, as those who chose not to answer specific questions might have distinct characteristics compared to participants who provided complete responses. However, it is important to note that the omitted questions were not specific to particular topics but rather random inquiries and thus were less likely to significantly influence the overall outcomes of the regression analyses.

### 3.2 Measures

#### 3.2.1 Dependent variables

*Intentions to change to plant‐based proteins, partially or entirely*. To measure intention in this study, we utilized two items measured on a 7‐point semantic differential scale. The first item (INT1) aimed to gauge individuals' tendency to reduce the consumption of animal protein sources.^4^ The second item (INT2) directly measures the intention to buy more plant‐based protein foods, assuming prices would remain the same. We recognize the importance of this dual‐item approach, as it allows us to capture the intention to reduce meat consumption and specifically embrace plant‐based protein alternatives. INT1 and INT2 were measured on a 7‐point scale of 1 (highly unlikely) to 7 (highly likely). To evaluate the internal consistency of our intention measure, we computed the alpha coefficient (*α*), which resulted in a value of .728, suggesting a satisfactory level of reliability.

#### 3.2.2 Predictors

##### Attitude

Six items were used to measure individual attitudes toward plant‐based protein foods. TPB defines attitudes as a person's overall evaluation or appraisal of behavior (Ajzen, 1991). In the context of plant‐based protein foods, attitudes can significantly influence individuals' intention to purchase and consume them. The six items used in this study cover various aspects of attitudes toward plant‐based protein foods, emphasizing negative evaluations. Each item captures a concern or perception commonly associated with plant‐based protein foods. For instance, ATT1 and ATT2 reflect concerns about the perceived level of processing and preservatives in these foods, while ATT3 and ATT4 address concerns about calorie content and sodium levels. ATT5 and ATT6 highlight concerns about the nutritional value and potential health risks associated with plant‐based protein foods. The high‐reliability coefficient (*α* = .89) indicates a strong internal consistency among these items (Table 1).

**TABLE 1:** Means, standard deviations, and reliabilities of the variable used in the study.

Constructs	Measurements	Mean (*SD*)	Loadings	Cronbach's alpha (*α*)
Intention (1 = highly unlikely; 7 = highly likely)				.728
INT1	Intention to reduce consumption of animal protein sources by at least 25% in the next 12 months	3.36 (1.87)	0.788	
INT2	Intention to buy more plant‐based protein foods if offered at the same price as their equivalent animal‐based food today	3.6 (2.08)	0.788	
Attitude (1 = strongly disagree; 7 = strongly agree)				.89
ATT1	Plant‐based protein foods are too processed	5.20 (1.55)	0.791	
ATT2	Plant‐based protein foods have too many preservatives	5.06 (1.55)	0.862	
ATT3	Plant‐based protein foods are too high in calories	4.24 (1.47)	0.808	
ATT4	Plant‐based protein foods are high in sodium	4.71 (1.51)	0.817	
ATT5	Plant‐based protein foods are low in nutritional value (e.g., iron, vitamin B12, etc.)	4.59 (1.49)	0.788	
ATT6	Plant‐based protein foods pose health concerns (i.e., allergies, etc.)	4.20 (1.71)	0.755	
Subjective norm (1 = strongly disagree; 7 = strongly agree)				
SN^a^	My food choices are largely influenced by family and friends	3.33 (1.5)		
Perceived behavioral control (1 = strongly disagree; 7 = strongly agree)				.729
Self‐efficacy				
PBC‐SE1	I do not know what plant‐based protein meals are made of	4.31 (1.76)	0.875	
PBC‐SE2	I do not know how to cook plant‐based protein meals	3.74 (1.73)	0.851	
PBC‐SE3	It is hard to incorporate plant‐based proteins into meals	3.61 (1.50)	0.655	
Availability (1 = strongly disagree; 7 = strongly agree)				.725
PBC‐AV1	It is difficult to find plant‐based meals in retail stores	3.64 (1.5)	0.790	
PBC‐AV1	It is difficult to find plant‐based meals in restaurants/cafes	4.14 (1.5)	0.940	
Sustainability and ethical concern	Factors considered important in choosing plant‐based protein foods (1 = not at all important; 7 = extremely important)			.802
SO1	Supporting the local economy (e.g., creating jobs in the community, etc.)	5.06 (1.69)	0.744	
SO2	Ethical concerns (animal welfare)	4.88 (1.97)	0.885	
SO3	Environmental concerns	5.13 (1.91)	0.902	
Meat attachment (1 = strongly disagree; 7 = strongly agree)				.65
MA1	Meat consumption is a habit difficult to overcome	4.40 (1.73)	0.403	
MA2	Meat is a necessary component of a “proper meal”	4.28 (1.88)	0.918	
MA3	Meat has an indispensable nutritional value.	4.62 (1.76)	0.910	
Past behavior (1 = no change; 5 = complete shift to a vegetarian diet)				
PB^a^	Change in the consumption of plant‐based protein foods in the past 12 months	1.48 (0.81)		
Sociodemographic factors^b^	Gender, marital status, age, parental status, education, household income, residential neighborhood, and region			

^*a*^ Single‐item variables.

^b^ Please refer to Table 3 for the concrete phrasing of sociodemographic variables in the questionnaire and descriptive statistics.

##### Subjective norm

This is a single‐item variable. A single item can yield valuable insights, notably when it strongly connects with the target construct (Bergkvist & Rossiter, 2007; Jang & Cho, 2022; Onwezen et al., 2019; Pieniak et al., 2010). Participants were asked to rate their level of agreement on a scale of 1 (strongly disagree) to 7 (strongly agree) in response to the statement, “Family and friends largely influence my food choices.” The influence of family and friends on individuals' attitudes and behaviors, including their dietary choices, can be significant (Lim & An, 2021). Therefore, it is crucial to understand the factors that drive purchase intention by capturing the impact of family and friends on decision‐making regarding plant‐based protein foods. Although using a single item to measure subjective norms may have limitations, its strong correlation with purchase intention supports its preliminary use in capturing the influence of family and friends on individuals' decision‐making regarding plant‐based protein foods.

##### Perceived behavioral control

Perceived behavioral control is a multifaceted construct incorporating internal (self‐efficacy) and external (perceived availability) factors. Self‐efficacy represents individuals' belief in their ability to successfully engage in a behavior, while perceived availability refers to the external factors that may act as barriers or facilitators to perform the behavior (Sparks et al., 1997; Vermeir & Verbeke, 2008). In this study, self‐efficacy (SE) was measured using three items: (PBC‐SE1) “I do not know how to cook plant‐based protein foods,” (PBC‐SE2) “It is hard to incorporate plant‐based proteins into meals,” and (PBC‐SE3) “I do not know what plant‐based protein meals are made of.” These items assess the individual's confidence and perceived competence in cooking and incorporating plant‐based proteins into their meals. Perceived availability, on the other hand, was measured using two items: (PBC‐AV1) “It is difficult to find plant‐based protein meals in retail stores” and (PBC‐AV2) “It is difficult to find plant‐based protein meals in restaurants and cafes.” These items capture the individuals' perceptions of the accessibility and availability of plant‐based meal options in their local retail stores and eating establishments. Both self‐efficacy and perceived availability contribute to individuals' sense of control over adopting plant‐based protein diets. Even if individuals possess high self‐efficacy, external barriers, such as limited availability or difficulty finding plant‐based protein meals, can still affect their perceived control over their behavior (Fresán et al., 2020). The items measuring self‐efficacy and perceived availability were rated on a scale of 1 (strongly disagree) to 7 (strongly agree) and yielded a Cronbach's alpha coefficient of .729 and .725.

##### Sustainability and ethical concern

This study utilized a set of three items to measure “Sustainability and ethical concern.” Participants were asked to rate the importance of various factors in choosing plant‐based protein foods on a 7‐point Likert scale. The measurement items included supporting the local economy (SO1), ethical concerns related to animal welfare (SO2), and environmental concerns (SO3). Each item demonstrated a significant and positive loading on the component matrix, indicating their relevance to the underlying construct. The reliability analysis yielded a high internal consistency (*α* = .802). This construct provides valuable insights into the motivations and values that underlie individuals' decisions to opt for plant‐based protein foods.

##### Meat attachment

Meat attachment has been recognized as a significant barrier in shifting toward plant‐based protein foods. Meat attachment is a distinct psychological construct that captures individuals' deeply ingrained habits, cultural beliefs, and personal preferences associated with meat consumption (Bryant & Barnett, 2019; Graça et al., 2019; Wang & Scrimgeour, 2021). Meat attachment is likely to persist and continue to influence individuals' purchase intentions, even if they have changed their past behavior or are considering embracing plant‐based protein foods. Therefore, considering meat attachment as a separate construct and examining its role alongside other TPB components provides a valuable opportunity to explore its unique influence on individuals' attitudes, beliefs, and intentions regarding plant‐based protein foods in Western societies. To assess participants' attachment to meat, we utilized a three‐item measurement scale with an acceptable level of consistency (*α* = .65). Participants were asked to express their attitude toward meat consumption using a 7‐point Likert scale, ranging from 1 (strongly disagree) to 7 (strongly agree). These measurement items were adapted from the work of Pohjolainen et al. (2015).

##### Past behavior

To measure buying behavior related to plant‐based protein foods, we used a single‐item measuring consumption patterns over the past 12 months. Several studies also used a single item to measure this construct (Contini et al., 2020; Seffen & Dohle, 2023). The measurement scale ranged from 1 to 5, allowing participants to indicate the degree of change in their consumption patterns, with 1 representing no change and 5 indicating a complete shift to a vegetarian diet. The intermediate values (2, “I have increased the consumption of plant‐based proteins by 25% or less”; 3, “I have increased the consumption of plant‐based proteins by 25% to 50%”; and 4, “I have increased the consumption of plant‐based protein by more than 50%”) provided finer distinctions to capture varying levels of increase in consumption. This item is aimed to capture participants' actual buying behavior regarding plant‐based protein foods within a specified timeframe. This retrospective assessment allows us to understand the extent of participants' past behavior related to plant‐based protein food consumption. It provides insights into the progress made in adopting such foods over the past year.

##### Sociodemographic and contextual variables

The study included gender, marital status, age, number of children, education level, household income, and context‐specific variables (residential neighborhood and region) to explore the association between sociodemographic variables and the intention toward sustainable diets. For example, based on nationally representative data from the 2015 Canadian Community Health Survey‐Nutrition, Valdes et al. (2021) found differences in food choices based on sociodemographic factors.

### 3.3 Statistical analyses

Descriptive and factor analyses were conducted using IBM SPSS software (version 28). The descriptive analysis provided an overview of the sample population's characteristics. Factor analysis was employed to uncover underlying factors influencing potential changes in dietary patterns among Canadian consumers.

The study utilized hierarchical regression analysis to examine the factors predicting intentions to change to plant‐based proteins, partially or entirely. Notably, researchers followed a specific order of entry when introducing these variables into the regression model (Choi & Johnson, 2019; Seffen & Dohle, 2023; Sharps et al., 2021). This sequential approach ensured a thorough examination of the factors influencing purchase intentions. Initially, demographic variables were included in the regression analysis. This step allowed for the control of potential confounding factors associated with participants' characteristics. Subsequently, the three key constructs of TPB were introduced into the analysis while controlling for the demographic variables. This allowed for understanding how these TPB constructs contribute to shaping purchase intentions while considering the influence of participants' demographic backgrounds. To enrich the analysis further, the variable “sustainability and ethical concern” was added, considering both the demographic variables and TPB constructs. This enabled an examination of how individuals' inclination toward sustainability influences their intentions to purchase plant‐based protein foods. Then the variable of meat attachment was incorporated into the analysis. This allowed for an exploration of how emotional or habitual attachment to meat‐based diets may affect individuals' willingness to adopt plant‐based protein alternatives. Finally, the variable related to past behavior was included in the analysis, considering the effects of TPB constructs, sustainability and ethical concern, meat attachment, and sociodemographic variables.

The correlation matrix provides insights into the relationships among the variables (Table 2). It is worth noting that the TPB variables, attitudes and perceived behavioral control, are reverse‐coded, with higher values indicating more negative perceptions. Consequently, the two TPB variables, attitude and perceived behavioral control, are directly correlated. The relationship between intention and the variable subjective norm is indirect, operating through the mediating role of attitudes. Moreover, the three additional variables, sustainability and ethical concern, meat attachment, and past behavior, show significant correlations with intention.

**TABLE 2:** Correlation matrix among the TPB and other key variables.

	Intention	Attitude	Subjective norm	Self‐efficacy	Perceived availability	Sustainability and ethical concern	Meat attachment
Attitude	−.200***						
Subjective norm	.020	.099***					
Self‐efficacy	−.241***	.251***	.299***				
Perceived availability	.137***	.077***	.255***	.382***			
Sustainability and ethical concern	.380***	.020	.0994***	−.002	.120***		
Meat attachment	−.566***	.263***	.0909***	.344***	−.012	−.2774***	
Past behavior	.481***	−.124***	.017	−.204***	.030	.170***	−.311***

*** *p* < .01.

## 4 RESULTS

### 4.1 Sociodemographic characteristics of study participants

Half of the respondents in the study identified as female (51%), and the majority (65%) reported being either married or in a common‐law relationship. In terms of age, the largest group fell in the 45–64 category (32.5%), followed by 25–34 years old (23%). Notably, a significant portion (64%) indicated they had no children in their households. Regarding education, a little over a third of the respondents (36%) had completed a university degree, certificate, or diploma. The largest group of participants (30%) reported an annual combined household income of $100,000 to $149,999. Finally, regarding residential location, 45% resided in urban areas and 51.5% lived in Central Canada. Overall, based on the available data, we can reasonably assert that our dataset is representative of the Canadian population, particularly in terms of gender, marital status, and residential area (urban vs. rural) (Table 3).

**TABLE 3:** Sociodemographic characteristics of study participants.

Variable	(%)
Gender (*n* = 1795)	
Male	46.5
Female	50.8
Other	0.9
Prefer not to answer	1.8
Marital status (*n* = 1796)	
Single	24.3
Married or common Law	65
Divorced or separated	8
Prefer not to answer	2.7
Age in years (*n* = 1792)	
18–24	3.7
25–34	23.4
35–44	20.1
45–64	32.5
≥65	20.2
Children in households (*n* = 1793)	
None	64.1
One	14
Two	13.4
Three or more	6.5
Prefer not to answer	2
Education status (*n* = 1795)	
(Some) high school diploma	11
Apprenticeship or other trades certificate	6.5
CEGEP or other non‐university certificate	25.6
University degree/certificate	35.7
Advanced University (graduate)	18.7
Prefer not to answer	2.6
Income^a^	
Less than $34,999	10.7
Between $35,000 and $49,999	11.8
Between $50,000 and $74,999	28.5
Between $100,000 and $149,999	30
$150,000+	18.9
Region(*n* = 1793)^b^	
Western Canada	12.2
Prairies	15.8
Central Canada	51.5
Atlantic Canada	20.5
Northern Canada	–
Neighborhood (*n* = 1783)	
Urban	45.3
Suburban	32.9
Rural	21.9

^**a**^ Wealth and income were defined as tertials of self‐reported per capita household income in Canada.

^**b**^ Western Canada: British Columbia; Prairies: Alberta, Saskatchewan, and Manitoba; Central Canada: Ontario and Quebec; Atlantic Canada: New Brunswick, Prince Edward Island, Nova Scotia, and Newfoundland and Labrador; and Northern Canada: Yukon, Northwest Territories, and Nunavut.

### 4.2 Food consumption behaviors of Canadian consumers

**FIGURE 2:**
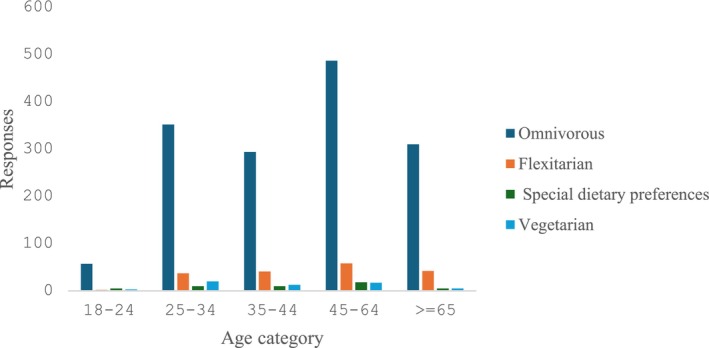
Relationship between age and food consumption habits.

Canadians in the study were asked to identify themselves in terms of a dietary type that best describes their current consumption habits: unrestricted omnivorous, flexitarian, special dietary preferences (other than vegetarian),^5^ and vegetarian (100% plant‐based). Most consumers in the sample (84%) identified as unrestricted omnivorous. Also, 10% of consumers in the sample reported being flexitarian, following a vegetarian diet but occasionally consuming meat or fish, and 3% of the study participants identified as vegetarian or vegan (Figure 2). On the other hand, about 3% of the consumers identified as having special dietary preferences.

**FIGURE 3:**
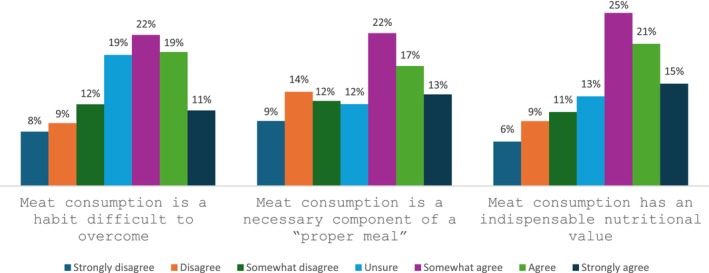
Consumer perceptions toward meat.

Figure 3 reflects the extent to which Canadians in the sample (dis)agree with the statements related to meat attachment. A significantly larger percentage of consumers somewhat to strongly agree with the statement, “Meat consumption is a habit difficult to overcome,” at 52%. Similarly, most Canadians in the sample (53%) somewhat to strongly agree with the statement, “Meat consumption is a necessary component of a ‘proper meal’.” Regarding the statement “Meat consumption has an indispensable nutritional value,” most consumers in the sample (61%) somewhat to strongly agree.

**FIGURE 4:**
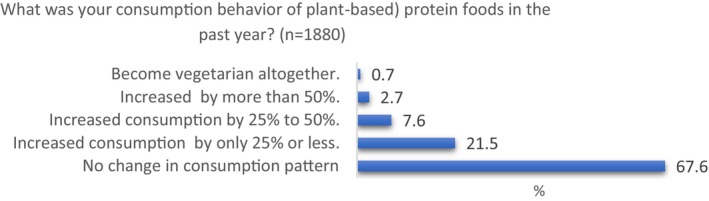
Consumption behavior of plant‐based protein foods in the past 12 months.

Figure 4 presents the consumption behavior of plant‐based protein foods over the past year. The respondents were asked to select from five options, ranging from “No change in consumption pattern” to “Become vegetarian altogether.” The results show that most of the consumers in the sample (68%) reported no change in their consumption behavior in the past year. About 22% reported a slight increase in consumption (i.e., 25% or less), while about 8% reported a moderate increase in consumption (i.e., 25% to 50%). Only around 3% of consumers reported a significant increase in consumption by more than 50%, and a very small percentage (about 1%) reported becoming vegetarian altogether.

### 4.3 Factors predicting intentions to change toward plant‐based protein diets

Table 4 displays the results of the hierarchical regression analysis conducted sequentially. Robust standard errors were employed to address potential heteroskedasticity in the data, ensuring more reliable standard errors and significance tests. The Variance Inflation Factor (VIF) was calculated to assess multicollinearity among the variables included in Models 1 to 5. All variables exhibited low VIF values, generally below 2.0, indicating minimal multicollinearity (see Appendix).

**TABLE 4:** Hierarchical regression analyses for the extended TPB.

Variables	Model 1	Model 2	Model 3	Model 4	Model 5
Gender (base = male)					
Female	.464*** (.05)	.468*** (.047)	.307*** (.046)	.174*** (.043)	.163*** (.04)
Other	.001 (.282)	−.097 (.213)	−.047 (.158)	−.207 (.137)	−.154 (.137)
Marital status (base = single)					
Married or common law	.086 (.069)	.06 (.062)	.022 (.058)	.012 (.053)	−.024 (.049)
Divorced or separated	−.032 (.111)	−.036 (.102)	.033 (.094)	−.015 (.084)	−.017 (.082)
Age	−.08*** (.023)	−.045** (.022)	−.037* (.02)	−.05*** (.018)	−.029* (.017)
Number of children	−.103*** (.025)	−.084*** (.024)	−.06*** (.023)	−.031 (.022)	−.022 (.02)
Education	.109*** (.021)	.088*** (.02)	.081*** (.019)	.052*** (.017)	.047*** (.016)
Household income	−.024 (.025)	−.017 (.022)	.022 (.021)	.006 (.02)	.008 (.019)
Neighborhood (base = urban)					
Suburban	−.121** (.057)	−.109** (.052)	−.088* (.049)	−.049 (.045)	−.016 (.043)
Rural	−.303*** (.067)	−.282*** (.06)	−.253*** (.056)	−.189*** (.052)	−.147*** (.049)
Region					
Prairies	−.114 (.094)	−.207** (.085)	−.15* (.08)	−.077 (.071)	−.077 (.067)
Central Canada	.009 (.076)	−.02 (.072)	−.01 (.067)	.027 (.06)	.014 (.057)
Atlantic Canada	−.118 (.087)	−.189** (.081)	−.179** (.076)	−.158** (.068)	−.166*** (.063)
TPB variables					
Attitude		−.123*** (.025)	−.133*** (.024)	−.053** (.021)	−.049** (.02)
Subjective norm		.031* (.016)	.017 (.015)	.027* (.014)	.018 (.013)
Perceived behavioral control					
Self‐efficacy		−.296** (.026)	−.276*** (.025)	−.147*** (.025)	−.106*** (.024)
Perceived availability		.27*** (.025)	.232*** (.024)	.178*** (.022)	.157*** (.021)
Extended TPB variables					
Sustainability and ethical concern			.337*** (.024)	.241*** (.023)	.21*** (.022)
Meat attachment				−.401*** (.026)	−.342*** (.024)
Past behavior					.344*** (.026)
_cons	.046 (.141)	−.132 (.142)	−.18 (.132)	−.051 (.12)	−.595*** (.122)
Observations	1557	1557	1557	1557	1557
*R*‐squared	.116	.235	.333	.445	.513
Δ*R*‐squared	–	.12	.10	.11	.07

*** *p* < .01,

** *p* < .05,

* *p* < .1.

The initial model, which included only sociodemographic variables, demonstrated a satisfactory fit with an *R*‐squared value of .111. Significant findings from this model revealed that gender influenced intentions, with females exhibiting a higher intention toward plant‐based protein foods than males (*p* < .001). Age also played a significant role, as younger individuals were more inclined toward plant‐based protein foods than older individuals (*p* < .001). The number of children displayed a significant effect, with individuals having fewer or no children showing higher intentions for plant‐based protein foods (*p* < .001). Moreover, education level had an impact, with higher education levels more likely aligned with an intention to purchase plant‐based protein foods (*p* < .001). In terms of neighborhood, individuals residing in suburban areas (*p* < .05) and rural areas (*p* < .001) demonstrated lower intentions compared to those living in urban areas.

In Model 2, which included both the TPB variables and the sociodemographic variables, the *R*‐squared value increased to .235 (*p* < .001), and the TPB variables explained an additional 12% of the variance in intentions to have dietary changes. Several sociodemographic variables from Model 1 maintained their significant effects on purchase intentions. The female category, number of children, education, and rural neighborhood remained substantial predictors (*p* < .001). Age also retained its relevance in predicting intentions, although its significance slightly diminished. With the introduction of the TPB variables in Model 2, new significant findings emerged. The attitude variable (reverse‐coded) showed a statistically significant coefficient (*p* < .001), indicating that a negative attitude was associated with lower intentions to adopt plant‐based protein diets. The subjective norm variable also demonstrated marginal significance, suggesting that a stronger influence from family and friends was associated with intentions to change toward plant‐based protein diets. Furthermore, the coefficients for the two constructs related to the perceived behavioral control (reverse‐coded) were statistically significant. Self‐efficacy demonstrated a negative relationship with intentions, suggesting that lower self‐efficacy led to lower intentions to change dietary patterns. Perceived availability, another component of perceived behavioral control, positively impacted intentions, indicating that individuals who perceive difficulty in finding plant‐based protein meals in retail stores, restaurants, and cafes are more likely to express higher intentions to adopt these dietary patterns.

Model 3 builds upon the findings of Model 2 by introducing the sustainability and ethical concern variable, which further enhances the understanding of purchase intentions for plant‐based protein foods. Including sustainability and ethical concern in Model 3 results in a notable increase in the *R*‐squared value to .333, explaining an additional 10% of the variance in purchase intentions while accounting for demographic variables and the TPB constructs. Consistent with the previous models, gender, number of children, education, and rural neighborhood remain significant predictors of purchase intentions (*p* < .001). Interestingly, the regional variables Prairies and Atlantic Canada show negative correlations with intentions to change to plant‐based protein diets compared to individuals living in Western Canada.

Regarding the TPB variables, the subjective norm becomes statistically insignificant, while attitude and the two perceived behavioral control constructs remain highly significant (*p* < .001). Introducing the sustainability and ethical concern variable yields a significant finding (*p* < .001), indicating that individuals with a stronger sustainable orientation exhibit higher intentions to purchase plant‐based protein foods.

Model 4 expands on the previous models by incorporating the meat attachment variable into the analysis of purchase intentions for plant‐based proteins. With an increased *R*‐squared value of .445, Model 4 demonstrates that meat attachment contributes an additional 11% to explaining variance in purchase intentions when controlling for demographic variables, TPB constructs, and sustainability and ethical concern. Consistent with the previous models, gender, education, and rural neighborhood remain significant predictors of purchase intentions (*p* < .001) but with a reduced effect size. Among the regional variables, Atlantic Canada demonstrates a negative correlation with intentions to change dietary patterns to plant‐based proteins, indicating lower purchase intentions than individuals living in Western Canada. Regarding the TPB variables, attitude, subjective norm, and self‐efficacy are significant predictors, emphasizing their importance in shaping purchase intentions. The sustainability and ethical concern variable demonstrates a strong positive effect on purchase intentions. Introducing the meat attachment variable yielded a highly significant association (*p* < .001), suggesting that individuals with a stronger attachment to meat express lower preferences to change to plant‐based proteins.

Model 5 represents the final model and incorporates the additional variable of past behavior, providing further insights into intentions toward plant‐based proteins. With an *R*‐squared value of .513, Model 5 indicates that including past behavior contributes an additional 7% to explaining variance in purchase intentions, controlling for demographic variables, TPB constructs, sustainability and ethical concern, perceived availability, and meat attachment. Consistent with previous models, gender, education, and rural neighborhood remain significant predictors of purchase intentions (*p* < .001). Similarly, Atlantic Canada exhibits a strong negative correlation with intentions toward plant‐based proteins compared to Western Canada. Among the TPB variables, attitude and self‐efficacy continue to be significant predictors. Notably, the perceived availability, sustainability, and ethical concern variables maintain their strong positive effect (*p* < .001). Conversely, the meat attachment variable significantly and negatively impacts intentions (*p* < .001). Lastly, the newly introduced variable of past behavior yields a highly significant and positive coefficient (*p* < .001), indicating that individuals with a history of engaging in behaviors related to plant‐based protein foods are more likely to express higher intentions to change toward such products.

## 5 DISCUSSION

### 5.1 The role of TPB variables in predicting intentions to transition to plant‐based protein foods

While most study participants maintained their consumption patterns, a significant portion of Canadians in the sample changed their dietary choices. As predicted by TPB and consistent with several studies (Carfora et al., 2019; Vermeir & Verbeke, 2008; Wang & Scrimgeour, 2021), the hierarchical regression (Table 4) analyses revealed significant relationships between TPB variables and the intention to change dietary patterns. The main findings are discussed below.

#### 5.1.1 Attitude

The findings regarding the attitude variable align with the hypotheses derived from TPB. The hypothesis would suggest that individuals with negative attitudes toward plant‐based protein foods would exhibit lower intentions toward such foods. The specific concerns mentioned in the six attitude items reflect perceived disadvantages or negative beliefs about plant‐based protein foods. These concerns may stem from various factors, including societal influences, personal experiences, or beliefs about the nutritional value and overall quality of these foods. According to TPB, such negative attitudes can act as barriers, inhibiting individuals from developing positive intentions and embracing plant‐based protein dietary patterns. In the study context, individuals who agree with the statements about concerns related to plant‐based protein foods may have weighed the perceived drawbacks more heavily (Table 1), resulting in a negative attitude toward these dietary patterns. The findings support the TPB's proposition that attitudes are important determinant of intentions (Carfora et al., 2019; Dupont & Fiebelkorn, 2020; Sogari et al., 2021). The findings imply the need to provide accurate and evidence‐based information about the nutritional composition, safety, and health benefits of plant‐based protein options to dispel misconceptions and alleviate concerns.

#### 5.1.2 Subjective norm

The subjective norm construct measuring the influence of family and friends on food choices did not significantly affect intentions toward plant‐based protein foods. It suggests that in the context of this study and potentially within Western societies, the influence of family and friends may not play a substantial role in shaping intentions toward plant‐based proteins. However, although positively correlated and marginally significant in Model 2 and Model 4 (Table 4), the finding suggests social influence from close relationships may not be a prominent factor in driving intentions to change dietary patterns. Other studies indicate that the effect of subjective norm may vary across different behaviors (McEachan et al., 2011) or have a weaker or no impact on purchase intentions compared to the other variables in the TPB (Armitage & Conner, 2001; Menozzi et al., 2017). This finding suggests that the influence of subjective norm on individuals' intentions to adopt plant‐based protein diets may be less significant than the other TPB variables.

#### 5.1.3 Perceived behavioral control

The findings regarding the perceived behavioral control constructs align with the theoretical foundations of TPB. The negative relationship between self‐efficacy and intentions suggests that individuals with lower self‐efficacy, perceiving challenges in understanding and cooking plant‐based protein meals and incorporating them into their diets, were less likely to express intentions to change their dietary patterns. The findings highlight the importance of adequate knowledge and skills to embrace and potentially increase the consumption of plant‐based proteins (Onwezen et al., 2021). Consistent with the TPB, lower self‐efficacy may undermine an individual's confidence in adopting new dietary habits and could act as a barrier to behavioral change. On the other hand, perceived availability focuses on the external factors that may facilitate or hinder behavior. The positive impact of perceived availability on intentions implies that individuals who perceive difficulty in finding plant‐based protein meals in retail stores, restaurants, or cafes are more likely to express higher intentions to change dietary patterns. This finding may seem counterintuitive at first, but it is not surprising. Those who perceive plant‐based protein options as less available are likely the ones who have a stronger desire and motivation to embrace more plant‐based protein diets. Consumers may make the most of the options if they have limited access to plant‐based protein sources. Thus, while they may face challenges in accessing these products, they still try to incorporate them into their diet to some degree. Also, difficulties in finding plant‐based protein sources may create a perception of scarcity and make consumers believe that these plant‐based proteins are hard to come by, placing a higher value on them and making conscious efforts to increase their consumption. Generally, the findings associated with the two constructs align with the TPB, which emphasizes that both internal factors, such as self‐efficacy, and external factors, such as perceived availability shape individuals' perceptions of control over consumption behavior.

### 5.2 Extended TPB variables: Sustainability and ethical concern, meat attachment, and past behavior

The study expanded the traditional TPB variables in predicting intentions to change to plant‐based proteins, partially or entirely, by incorporating sustainability and ethical concerns, meat attachment, and past behavior; they collectively accounted for approximately 28% of the intention variance.

Including sustainability and ethical concern yielded a significant and positive association with intentions, explaining an additional 10% of the variance beyond the TPB. Other studies also highlight the significance of sustainability in food choices (Bryant & Sanctorum, 2021; Graça et al., 2015; Kevany, 2020; Pandey et al., 2021). Our findings emphasize that individuals increasingly considering the broader implications of their food choices seek more sustainable alternatives. In the study, supporting the local economy, animal welfare, and environmental concerns were positively associated with dietary changes. These findings highlight the need for interventions and marketing strategies addressing such sustainability and ethical considerations.

Including the meat attachment construct provided valuable insights into its impact on intentions to change to plant‐based proteins, partially or entirely. The results showed a negative association between meat attachment and intentions, contributing an additional 11% to the variance in intentions toward plant‐based protein foods, controlling for demographic variables, TPB constructs, and sustainability and ethical concerns. The findings complement the TPB variables by shedding light on the emotional and habitual aspects of meat consumption. While the TPB focuses on cognitive factors, meat attachment captures the emotional connection and the perception of meat as an essential part of meals. It emphasizes the role of ingrained habits and deeply rooted preferences in influencing consumer intentions to shift toward plant‐based protein alternatives. Several studies also highlight that positive attitudes toward meat are a significant constraint in embracing a sustainable diet (Graça et al., 2019; Mackenzie & Shanahan, 2018; Malek et al., 2019; Shekarian & Mellat Parast, 2021). Meat appreciation may carry various symbolic meanings and sensory characteristics (Fox et al., 2021; Pohjolainen et al., 2015). Food neophobia is another factor attributed to the low uptake of plant‐based protein sources (de Koning et al., 2020; Losada‐Lopez et al., 2021). Canadian identity, for many, may be integrated into widely promoted yet unexamined notions of Canadians being a meat‐producing and consuming nation (Hannan, 2023). Understanding the influence of meat attachment is crucial for developing effective interventions and marketing strategies promoting plant‐based protein foods (Bryant & Barnett, 2019; Graça et al., 2019; Wang & Scrimgeour, 2021). Strategies that solely focus on providing information about the benefits of plant‐based protein diets may not be sufficient if they fail to address the emotional attachment and perceived importance of meat.

Adding past behavior as a variable in this study provided valuable insights into its relationship with intentions for plant‐based protein foods. Consistent with previous research (Bacon & Krpan, 2018; Contini et al., 2020; Koklic et al., 2019; Zhang et al., 2019), the results revealed a strong positive association between past behavior and intentions. This means that individuals who have previously engaged in behaviors related to plant‐based protein foods are more likely to express higher intentions to change toward such products. The study measured past behavior using a self‐report item that assessed consumption patterns over the past 12 months. While some studies have suggested that past behavior does not affect purchase intentions (Cheng et al., 2005; Seffen & Dohle, 2023), our findings, as shown in Table 4, demonstrate that including past behavior contributed an additional 7% to the variation in purchase intentions. The significance of including past behavior lies in capturing individuals' experiences and behaviors, influencing their future intentions to purchase plant‐based protein foods. This finding complements the TPB variables by introducing a temporal dimension into the analysis. By understanding the role of past behavior, interventions and strategies can be developed to target individuals who have already shown an inclination toward plant‐based protein consumption, facilitating their transition and encouraging further consumption of sustainable diets.

### 5.3 The role of sociodemographic and contextual factors in predicting intentions to transition to plant‐based protein diets

The study identified two sociodemographic variables—gender and education—and two contextual variables—residential neighborhood and region—that consistently predicted intentions toward plant‐based protein diets among Canadian consumers.

First, females were more likely to purchase plant‐based protein foods than males. This finding is consistent with most previous research highlighting women's greater receptiveness to dietary changes and interest in plant‐based protein options (Bryant & Sanctorum, 2021; Kevany, 2020; Pandey et al., 2021). The finding suggests that gender differences shape individuals' intentions and the need for tailored interventions and marketing strategies among different gender groups.

Second, education level emerged as a significant predictor, with higher education levels associated with a greater intention to change toward plant‐based protein foods. This finding aligns with most earlier studies and suggests that education raises individuals' awareness and knowledge about the benefits of plant‐based protein diets (Havermans et al., 2021; Kirbiš et al., 2021; Niva & Vainio, 2021). This finding underscores the importance of educational initiatives and information campaigns to promote the consumption of plant‐based protein foods (Bianchi et al., 2022; Jang & Cho, 2022).

The study also found that residential neighborhoods significantly influenced intentions toward dietary changes to plant‐based proteins, a variable rarely explored in previous studies. Individuals living in rural areas demonstrated lower intentions toward plant‐based protein foods than those in urban areas. This finding highlights the influence of the local environment on individuals' dietary intentions. Factors, such as cultural dietary traditions and the availability and accessibility of plant‐based options, may contribute to this difference (Fresán et al., 2020). Addressing barriers and creating supportive environments in rural areas could promote plant‐based protein foods.

Furthermore, regional differences were observed, particularly in Atlantic Canada; a negative correlation between intentions toward plant‐based proteins and residents in the region was noted compared to those in Western Canada. This suggests that individuals in Atlantic Canada may have stronger attachments to animal‐based proteins or different cultural and regional influences that make them less inclined to reduce their intake. It also implies that the availability and development of plant‐based protein options may vary across regions. Understanding these regional variations is important for tailoring interventions and considering cultural and geographical contexts in promoting plant‐based dietary changes.

### 5.4 Study limitations and future directions

While the study provides novel insights into the factors predicting sustainable dietary choices related to plant‐based proteins and is one of the few national‐based studies conducted in Canada, caution must be taken when interpreting the results. First, the study measured behavioral intention rather than actual behavior, suggesting a need for future research to explore the alignment between intention and consumption behavior. Second, our study specifically focused on individual‐level factors predicting intentions; we encourage future research to explore how the external factors highlighted in our conceptual model (Figure 1) influence individuals' intentions and behaviors concerning plant‐based protein consumption. Also, future studies could explore intrinsic attributes, such as taste, texture, and aroma differences of specific plant‐based and animal‐based protein sources, to gain additional insights into the barriers to promoting plant‐based protein diets. Finally, while our findings are statistically robust, the practical implications of some effects may be modest.

## 6 CONCLUSIONS

This study examined the factors predicting Canadians' intentions to transition to plant‐based protein diets, partly or entirely. The study confirms the relevance of the TPB constructs in understanding intentions toward plant‐based protein foods. Specifically, attitudes, self‐efficacy, and perceived availability consistently emerged as significant predictors of intentions, although they explained only 12% of the variation. To achieve these changes in dietary patterns, it is necessary to focus on enhancing positive attitudes and self‐efficacy beliefs, which have been identified as key influencers of individuals' intentions toward plant‐based protein consumption. Improving the availability, visibility, and promotions of plant‐based protein alternatives in retail stores and restaurants is crucial for encouraging consumer intentions and facilitating behavioral change.

In the extended TPB model, the “sustainability and ethical concern” variable predicted 10% of the variation in intentions toward plant‐based protein diets. Integrating environmental and ethical messaging into marketing strategies can promote plant‐based protein consumption by appealing to individuals' values and concerns. Furthermore, individuals' past behavior regarding plant‐based protein consumption strongly influences their intentions. Conversely, the negative impact of meat attachment on intentions underscores the need to address psychological and emotional factors associated with meat consumption. Strategies highlighting the benefits of plant‐based protein foods can reduce resistance and promote sustainable dietary choices.

The findings also demonstrate the importance of demographics (such as gender and education levels) and contextual factors in shaping dietary preferences. Living in rural areas was associated with a lower intention toward plant‐based proteins, potentially due to cultural significance and limited availability of plant‐based options. Regional differences were observed in intentions toward plant‐based protein foods, with individuals from Atlantic Canada showing less intention than those from Western Canada. These demographic and contextual factors should be considered when developing targeted interventions to promote sustainable diets.

In conclusion, a multifaceted approach is recommended to achieve the desired transition to sustainable protein diets, encompassing strategies to enhance positive attitudes, self‐efficacy, and availability, emphasizing sustainability and ethical concerns, and addressing psychological factors associated with meat consumption. Practical implications include targeted marketing strategies, educational campaigns, and interventions tailored to specific demographic and contextual considerations. While this study primarily focuses on Canada, its findings broadly apply to other countries. This wide‐reaching relevance is attributed to the comprehensive analysis undertaken within the extended TPB framework and the study's consideration of a wide range of well‐established factors and individual‐level variables. These aspects contribute to our understanding of intentions as the call for transitioning toward sustainable consumption grows. Furthermore, the study addresses the global gap in the intersection of the extended TPB and plant‐based proteins, which is rarely explored in the literature.

## AUTHOR CONTRIBUTIONS

**Gumataw Kifle Abebe:** Conceptualization (lead); data curation (lead); formal analysis (lead); funding acquisition (supporting); methodology (lead); project administration (equal); resources (lead); software (lead); supervision (lead); validation (lead); visualization (lead); writing – original draft (lead); writing – review and editing (lead). **Mariam R. Ismail:** Formal analysis (supporting); investigation (supporting); methodology (supporting); project administration (supporting); visualization (equal); writing – original draft (supporting); writing – review and editing (supporting). **Kathleen Kevany:** Conceptualization (supporting); funding acquisition (lead); project administration (equal); resources (lead); supervision (supporting); writing – review and editing (supporting). **Hiwot Abebe Haileslassie:** Funding acquisition (supporting); project administration (supporting); resources (supporting); supervision (supporting); writing – review and editing (supporting). **Liam Young:** Writing – original draft (supporting). **Treasa Pauley:** Funding acquisition (lead); project administration (lead); writing – original draft (supporting).

## FUNDING INFORMATION

The study received funding from Protein Industries Canada as part of the “Plant Protein Atlantic: Exploring the Value of Plant Proteins in the Atlantic Region” project [No. CAP22.29].

## CONFLICT OF INTEREST STATEMENT

The authors confirm that no identifiable competing financial interests or personal relationships that could have influenced the work presented in this study.

## ETHICS STATEMENT

The studies involving human participants were reviewed and approved by the Dalhousie University Ethical Board. Written informed consent for participation was not required for this study in accordance with the national legislation and the institutional requirements.

## Data Availability

Data will be made available on request.

## Footnotes

^1^
A notable gap exists regarding establishing a universally accepted definition for plant‐based protein food, necessitating the need to provide a clear explanation for this term (Storz, 2022). In this study, plant‐based protein food refers to a food product that derives its protein content primarily or exclusively from plant sources and their derivates and “animal‐based protein foods” as diets primarily or exclusively comprising animal protein sources. Additionally, terms, such as “plant‐based proteins” or “plant‐based protein diets,” are often used interchangeably throughout the manuscript.

^2^
Please note that although our conceptual framework (Figure 1) incorporated external influences on dietary choices, such as national food guides, public food provision, government subsidies, pandemics such as COVID‐19, and media drivers like advertisements and discounts, this study did not examine these relationships. Instead, we focused on individual‐level factors and their impact on intentions to adopt plant‐based protein foods. Future research is warranted to investigate how these external factors influence individuals' intentions and behaviors concerning plant‐based protein consumption.

^3^
The original questionnaire can be obtained upon request from the corresponding author.

^4^
Please note that this question in itself does not necessarily imply a direct substitution with plant‐based protein and should be interpreted alongside the second question.

^5^
This includes lacto‐ovo vegetarian that excludes animal‐based foods; lacto‐vegetarian that avoids animal based‐foods and eggs; ovo‐vegetarian that excludes animal‐based foods except for eggs; and pesco‐vegetarian, those incorporating seafood into an otherwise vegetarian diet.

## VARIANCE INFLATION FACTOR (VIF) VALUES FOR MULTICOLLINEARITY ASSESSMENT

### 1

**Table:**

Variable	VIF	1/VIF
Gender		
Female	1.16	0.86569
Other	1.03	0.974561
Marital status		
Married or common law	1.72	0.582978
Divorced or separated	1.36	0.736199
Age	1.24	0.809147
Number of children	1.18	0.847754
Education	1.19	0.840941
Household income	1.49	0.673107
Neighborhood		
Suburban	1.23	0.81224
Rural	1.27	0.788742
Region		
Prairies	1.96	0.508976
Central Canada	2.57	0.389394
Atlantic Canada	2.18	0.459664
TPB variables		
Attitude	1.17	0.857286
Subjective norm	1.13	0.882949
Perceived behavioral control	1.32	0.758562
Extended TPB variables		
Sustainability and ethical concern	1.22	0.821984
Meat attachment	1.45	0.687543
Past behavior	1.16	0.8617
Mean VIF	1.42	
